# The role of hippocampal niche exosomes in rat hippocampal neurogenesis after fimbria–fornix transection

**DOI:** 10.1074/jbc.RA120.015561

**Published:** 2021-01-22

**Authors:** Xiang Cheng, Wen Li, Rongzhen Zhao, Haoming Li, Jianbing Qin, Meiling Tian, Xinhua Zhang, Guohua Jin

**Affiliations:** 1 Department of Human Anatomy, Institue of Neurobiology, Medical School of Nantong University, Nantong, Jiangsu, PR China; 2 Key Laboratory Neuroregeneration of Jiangsu and Ministry of Education, Co-innovation Center of Neuroregeneration, Nantong University, Nantong, Jiangsu, PR China

**Keywords:** exosome, neural stem cell, hippocampus, neurogenesis, neurodifferentiation, CNS, central nervous system, EVs, extracellular vesicles, FFT, fimbria–fornix transection, NSCs, Neural stem cells, SGZ, subgranular zone, TFs, transcription factors

## Abstract

Exosomes transfer signaling molecules such as proteins, lipids, and RNAs to facilitate cell–cell communication and play an important role in the stem cell microenvironment. In previous work, we demonstrated that rat fimbria–fornix transection (FFT) enhances neurogenesis from neural stem cells (NSCs) in the subgranular zone (SGZ). However, how neurogenesis is modulated after denervation remains unknown. Here, we investigated whether exosomes in a denervated hippocampal niche may affect neurogenesis. Using the FFT rat model, we extracted hippocampal exosomes and identified them using western blots, transmission electron microscopy (TEM), and nanoparticle size measurement. We also used RNA sequencing and bioinformatic analysis of exosomes to identify noncoding RNA expression profiles and neurogenesis-related miRNAs, respectively. RNA sequencing analysis demonstrated 9 upregulated and 15 downregulated miRNAs. miR-3559-3P and miR-6324 increased gradually after FFT. Thus, we investigated the function of miR-3559-3P and miR-6324 with NSC proliferation and differentiation assays. Transfection of miR-3559-3p and miR-6324 mimics inhibited the proliferation of NSCs and promoted the differentiation of NSCs into neurons, while miR-3559-3p and miR-6324 inhibitors promoted NSC proliferation and inhibited neuronal differentiation. Additionally, the exosome marker molecules CD9, CD63, and Alix were expressed in exosomes extracted from the hippocampal niche. Finally, TEM showed that exosomes were ∼100 nm in diameter and had a “saucer-like” bilayer membrane structure. Taken together, these findings suggest that differentially expressed exosomes and their related miRNAs in the denervated hippocampal niche can promote differentiation of NSCs into neurons.

Neural stem cells (NSCs) are pluripotent and have the capability of self-renewal. They exist in the developing brain and almost all mammalian adult nervous systems (1). NSCs contribute to neurogenesis by generating neurons, astrocytes, and oligodendrocytes, the three major cell types in the central nervous system (CNS). Manipulating their destiny has therapeutic promise in the treatment of adult brain injury and neurodegenerative disease (2, 3). The factors affecting NSC differentiation into neurons are very complex. Activation of quiescent adult NSCs is coordinated by the interaction between external stimuli from the niche and internal factors such as transcription factors (TFs), signaling pathways, epigenetics, and metabolism. This interaction initiates the intracellular regulation process to promote quiescent NSC exit from the G0 phase into a new cell cycle (4). Our previous studies have shown that NSCs transplanted into the subgranular zone (SGZ) of fimbria–fornix transection (FFT) brains exhibit strong proliferation and neuronal differentiation characteristics and formed a new neurogenic niche (5, 6).

The neurogenic niche is a unique and specialized microenvironment that promotes NSC proliferation and differentiation toward the neuronal lineage. Intercellular communication is particularly important in the dynamic regulation of homeostasis and plasticity in adult neurogenesis. Various signaling molecules, including numerous cytokines, neurotransmitters, and hormones, play an important role (7, 8, 9). In addition, extracellular vesicles (EVs), particularly exosomes, may play a role (10). Exosomes transmit information by carrying mRNA, miRNA, protein, and lipid between cells (11) and possess the ability to regulate biological processes of receptor cells. They may play an important role in various stem cell microenvironments. The expression of exosomal miRNAs is dysregulated in cancer-associated fibroblasts (12). PTEN is an important tumor suppressor, and its expression in primary tumor cells is closely related to astrocyte-derived exosomes in the brain microenvironment (13). Thus, exosomes are a major player in the intercellular communication network (14), as they can facilitate cross talk between cells located in distant locations (15).

Our previous studies have found that neurogenesis occurs in the hippocampal dentate gyrus after transection of the cholinergic innervation projecting from the basal forebrain to the hippocampus, which indicates that the denervated hippocampus provides an appropriate microenvironment for NSC survival and neuronal differentiation (16, 17). This study examines exosomes released by neurons, oligodendrocytes, astrocytes, and microglia as well as gene transcriptome expression profiles in the hippocampal niche after FFT to identify the specific mechanism involved in the proliferation and differentiation of hippocampal NSCs.

## Results

### Identification of exosomes in the hippocampal niche after FFT

**Figure 1:**
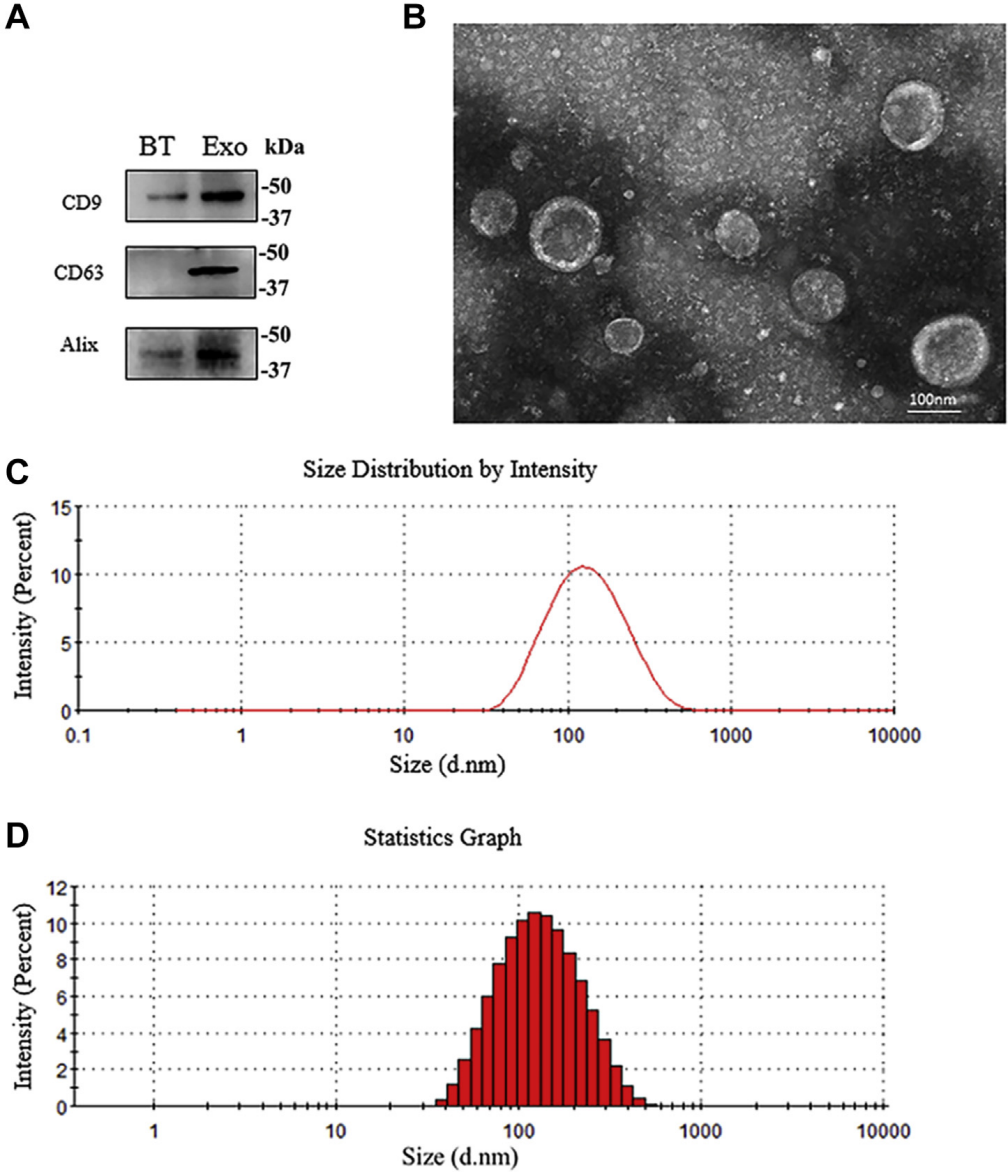
**Identification of hippocampal tissue exosomes.***A*, western blots showed that the marker proteins CD9, CD63, and Alix were expressed in hippocampal exosomes. *B*, transmission electron microscopy after negative staining with Teflon showed a clear “saucer-like” double-layer membrane structure. *C*, exosome size distribution. *D*, exosome size bar chart.

Western blots showed expression of exosome marker molecules CD9, CD63, and Alix (Fig. 1*A*). Exosome samples were negatively stained by Teflon; transmission electron microscopy showed that the extracted vesicles have a typical “saucer-like” double-layer membrane structure approximately 100 nm in diameter, a classical exosome characteristic (Fig. 1*B*). ZETASIZER Nano series-Nano-ZS analysis showed an average vesicle size of 105.3 ± 6.2 nm. Main peak vesicle size was 146.5 nm. The vesicle distribution coefficient ranged between 0.08 and 0.7, indicating that the hippocampal niche excretes exosomes after FFT (Fig. 1, *C*–*D*).

### Differential miRNA profiles of exosomes in the hippocampal niche after FFT

**Figure 2:**
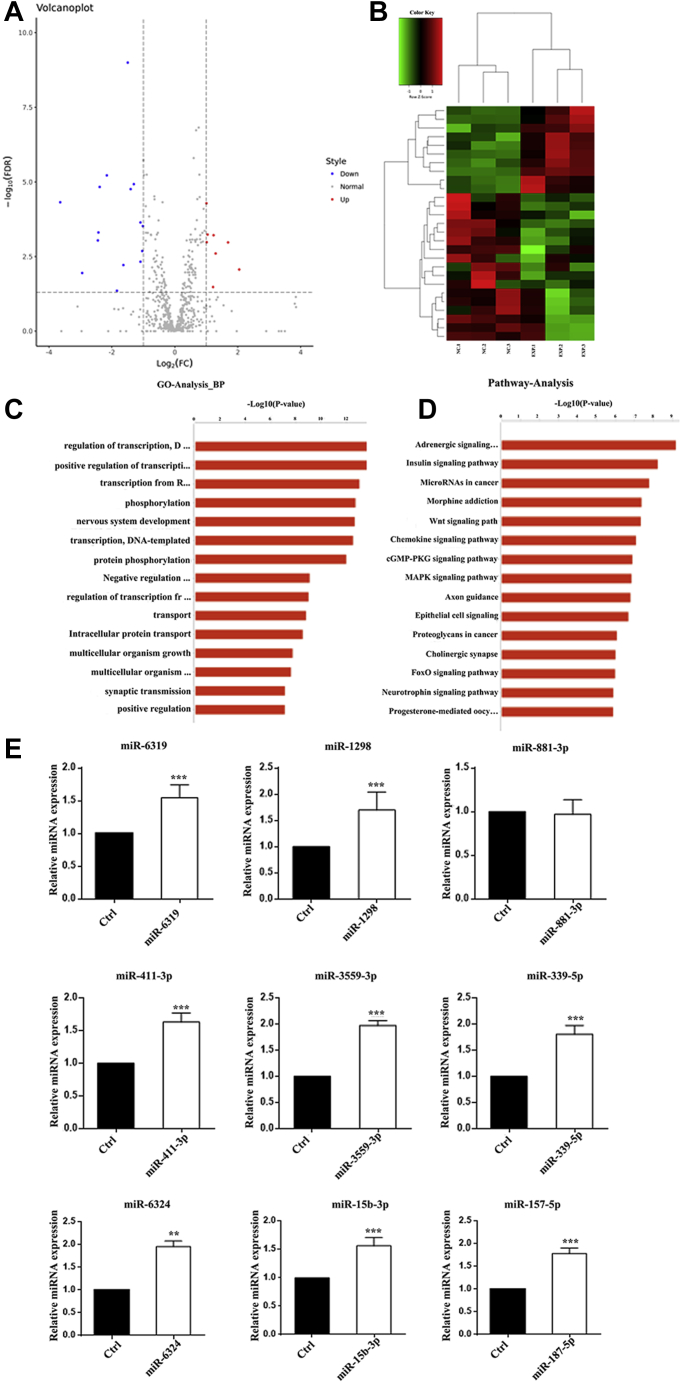
**RNA sequence analysis of exosomes in the hippocampus niche after FFT.***A*, differentially expressed miRNA volcano map, red for upregulation and blue for downregulation; *B*, hierarchical clustering analysis of differentially expressed miRNAs, red for upregulation and green for downregulation. *C*, differentially expressed miRNA gene ontology analysis showed that these differential miRNAs are mainly concentrated in biological processes such as transcriptional regulation, phosphorylation, and nervous system development. *D*, differentially expressed miRNA pathway analysis showed that these miRNAs are mainly involved in the insulin signaling, Wnt signaling, and cGMP-PKG signaling pathways. *E*, real-time PCR results of differentially expressed miRNAs (∗∗*p* < 0.01, ∗∗∗ *p* < 0.001).

RNA sequencing of exosomes showed 24 differentially expressed miRNAs. Among these, 9 were upregulated and 15 were downregulated in the FFT group (Fig. 2, *A*–*B*). Gene ontology analysis showed that these differential miRNAs are mainly concentrated in biological processes such as transcriptional regulation, phosphorylation, and nervous system development (Fig. 2*C*). KEGG pathway enrichment analysis showed that the differentially expressed miRNAs in the denervated hippocampal niche were mainly enriched in pathways involved in the insulin signaling, Wnt signaling, and cGMP-PKG signaling pathways (Fig. 2*D*). A total of nine exosomal miRNAs in each group with log fold-changes ≥1.5 were selected to further validate the RNA sequencing results. Real-time PCR results revealed that miR-411-3p, miR-3559-3p, miR-6324, miR-1298, miR-15b-3p, miR-187-5p, miR-6319, and miR-339-5p were upregulated compared with the exosomes from the normal hippocampal niche (Fig. 2*E*).

### The expression of miR-3559-3P and miR-6324 in hippocampal niche exosomes

**Figure 3:**
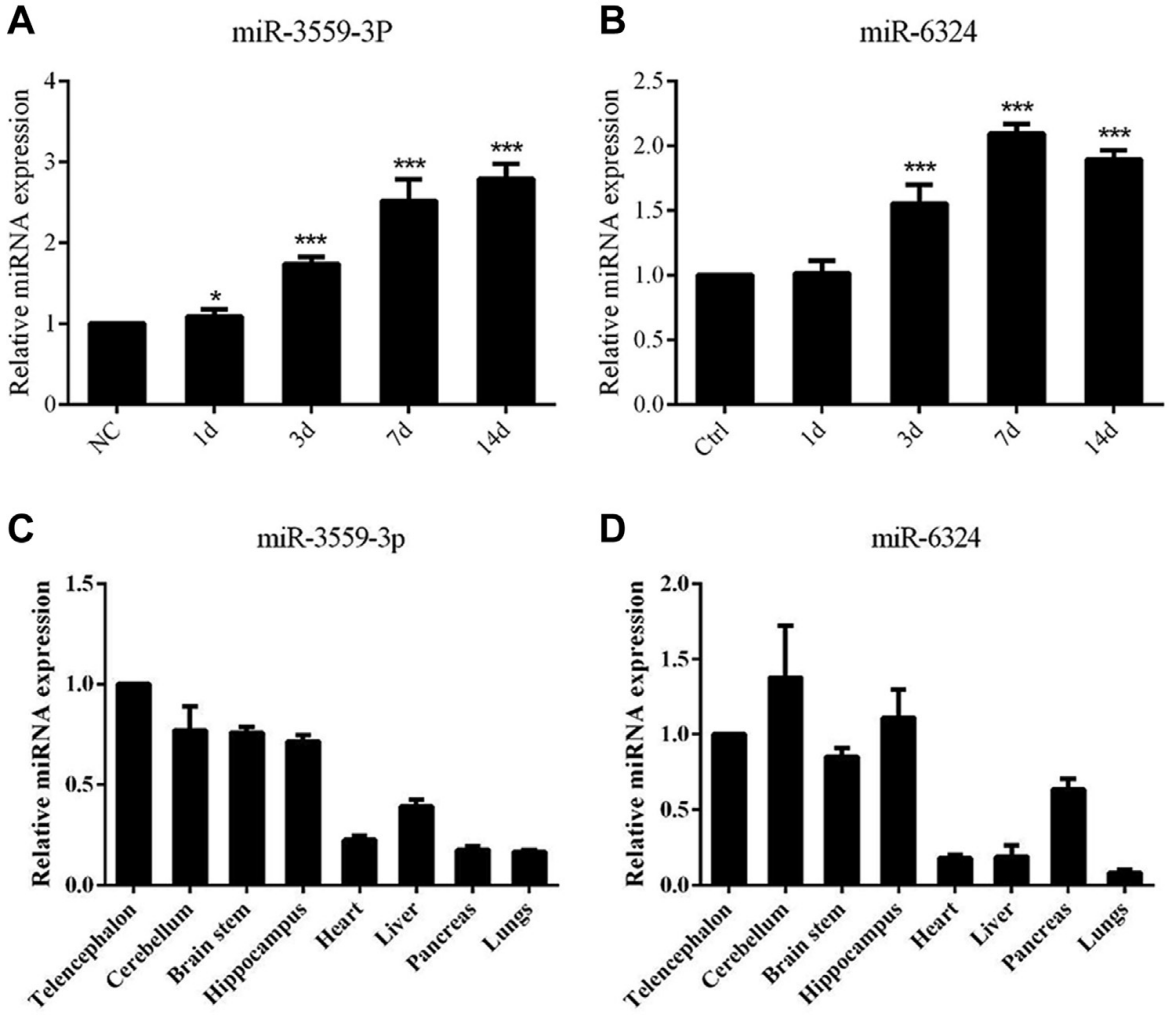
**Expression of miR-3559-3P and miR-6324 in the rat hippocampus.***A*, the expression level of miR-3559-3P and miR-6324 gradually increased after FFT; the expression level remained elevated after the seventh day (∗∗*p* < 0.01, ∗∗∗*p* < 0.001). *B*, the expression level of miR-6324 gradually increased after FFT; the expression level remained elevated after the seventh day (∗∗*p* < 0.01, ∗∗∗*p* < 0.001). *C*, real-time PCR results showed that miR-3559-3P was expressed greater in the brain than other tissues, and it was highly expressed in the hippocampus. *D*, real-time PCR results showed that miR-6324 was expressed greater in the brain than other tissues, and it was highly expressed in the hippocampus.

Unlike protein-coding genes, miRNAs sometimes exhibit tissue specificity for their particular role. We examined the expression of nine exosomal miRNA in tissue derived from ectoderm, mesoderm, and endoderm by real-time PCR. The results showed that, among these miRNAs, miR-3559-3P and miR-6324 were expressed greater in brain derived from ectoderm than other tissues and they were highly expressed in the hippocampus (Fig. 3, *C*–*D*). Thus, miR-3559-3P and miR-6324 were selected for further study. RNA of hippocampus niche exosomes was examined at 1 day, 3 days, 7 days, and 14 days after cholinergic denervation. Real-time PCR showed that the expression of miR-3559-3P and miR-6324 gradually increased after denervation and was greatest at 7 days after FFT (Fig. 3, *A*–*B*).

### The effect of exosomes from hippocampus niche on hippocampal NSC

**Figure 4:**
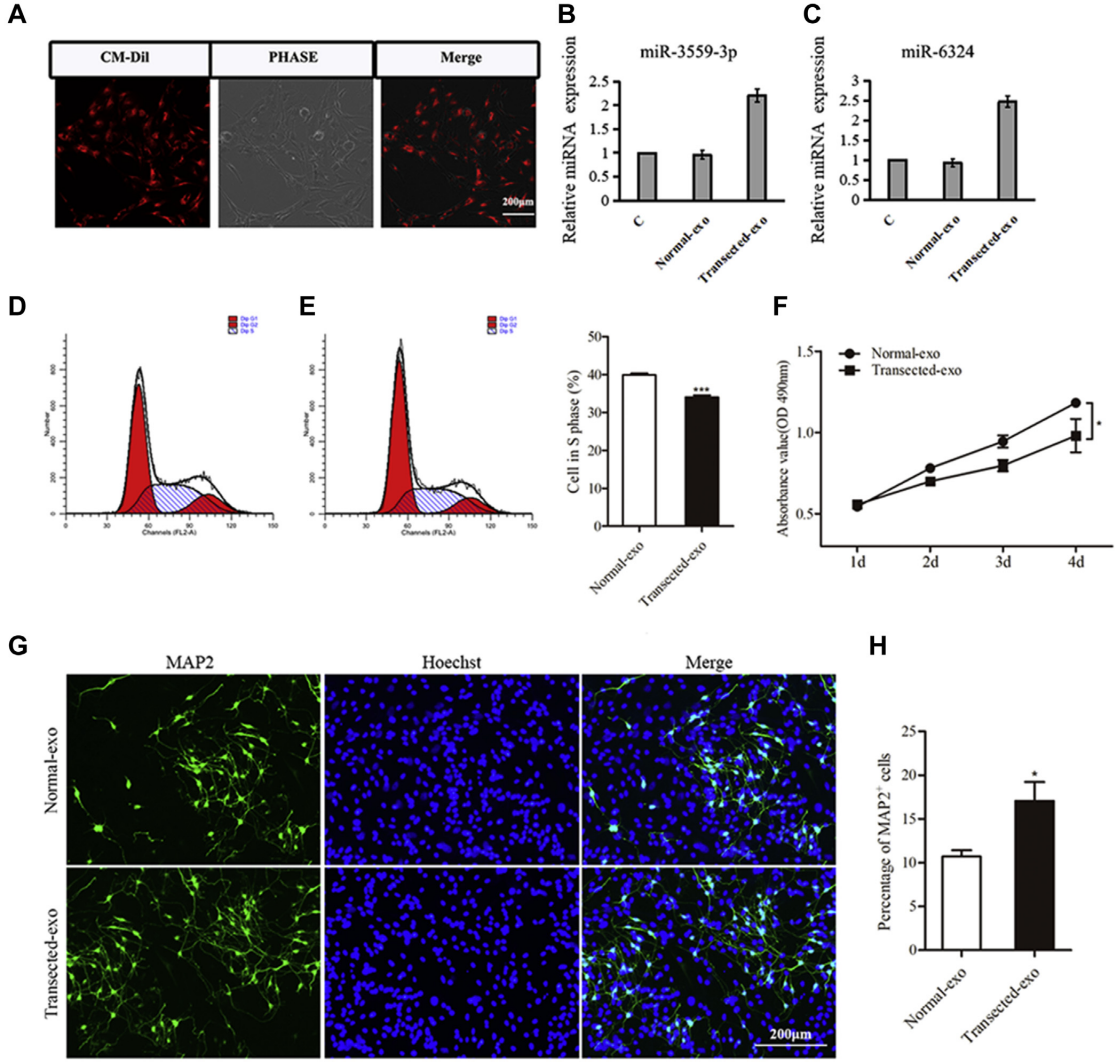
**Coculture the exosomes from hippocampus niche with hippocampus NSCs.***A*, CM-DiI positive NSCs after coculture with CM-DiI labeled exosomes. *B*–*C*, the expression level of miR-3559-3p and miR-6324 in hippocampus NSCs after cocultured with the exosomes from hippocampus. *D*–*F*, the proliferation of NSCs cocultured with transected-exo significantly decreased, compared with the NSCs cocultured with normal-exo. *G*–*H*, the MAP-2 positive cells increased in differentiation assay after cocultured with transected-exo.

To verify the role of exosomes in neurogenesis in the deafferented hippocampal niche, the CM-Dil labeled exosomes were cocultured with hippocampal NSCs. After 24 h, the proportion of CM-Dil positive NSCs was 94.1% ± 2.4%, implying that the exosomes could be taken up by NSCs (Fig. 4*A*). Real-time PCR showed that the expression of miR-3559-3P and miR-6324 in hippocampus NSCs cocultured with transected exosomes was higher than that in hippocampus NSCs cocultured with normal exosomes collected from normal hippocampus niche (Fig. 4, *B*–*C*). FCS showed that the proportion of hippocampus NSCs cocultured with transected exosomes in S phase was lower than that of hippocampus NSCs cocultured with normal exosomes (Fig. 4, *D*–*F*). In differentiation assay, the percentage of MAP-2 positive cells differentiated from hippocampus NSCs cocultured with transected exosomes was higher than that differentiated from hippocampus NSCs cocultured with transected exosomes (Fig. 4, *G*–*H*). Altogether, it suggests that exosomes from deafferented hippocampal niche could be taken up by hippocampal NSC promoting the expression of miR-3559-3P and miR-6324 and inducing differentiation toward neurons.

### The effect of miR-3559-3P and miR-6324 overexpression on hippocampal NSC proliferation and differentiation

**Figure 5:**
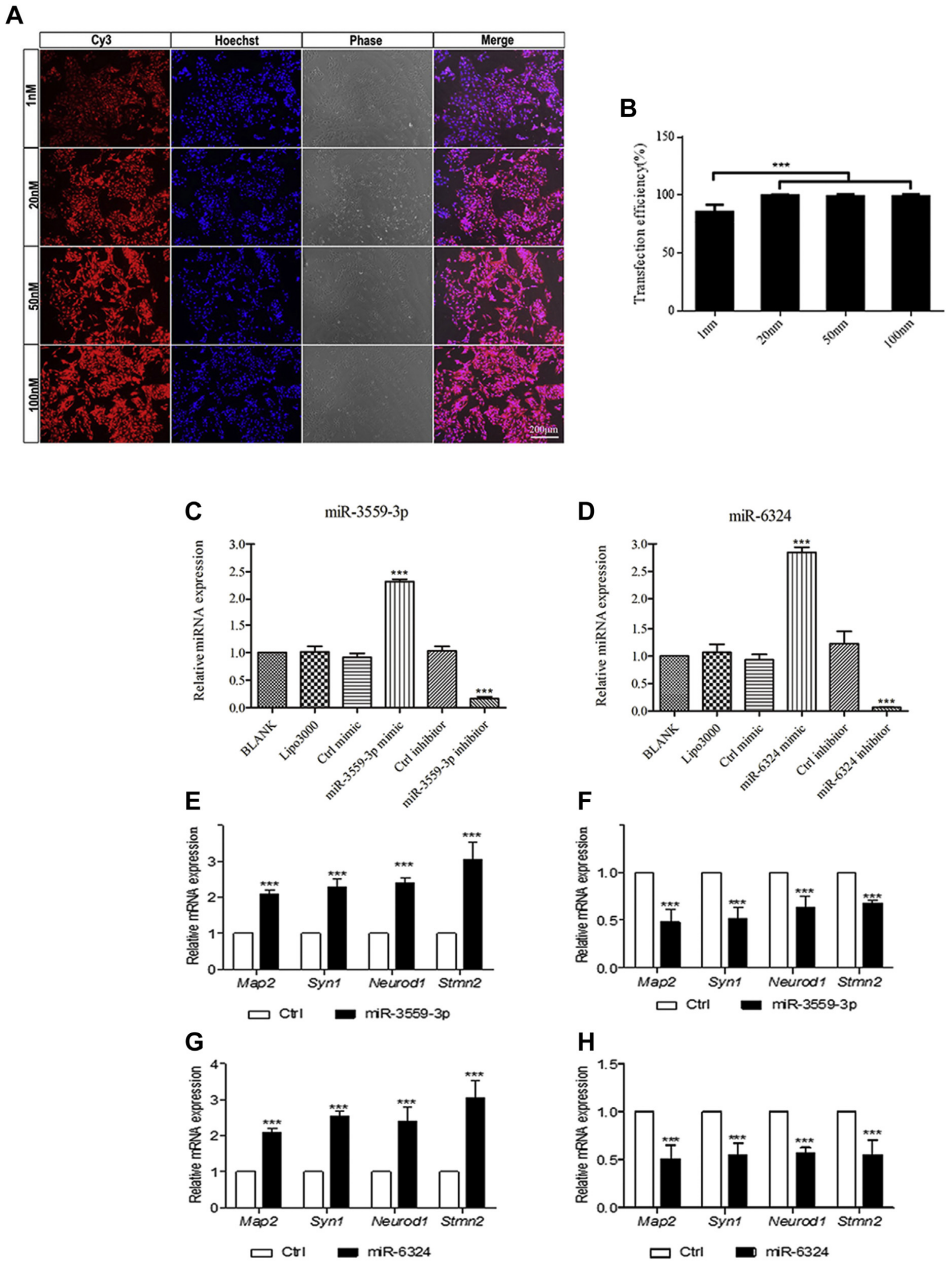
**Gain and loss of NCS function.***A*–*B*, transfection efficiency reached 100% with each transfection concentration (∗∗∗*p* < 0.001). *C*–*D*, statistical chart of expression and inhibition efficiency of miR-3559-3p and miR-6324 (∗∗∗ *p* < 0.001). *E*–*H*, statistical chart of the expression of neuron-specific molecules (∗∗∗*p* < 0.001).

To explore the role of miR-3559-3P and miR-6324 in the proliferation and differentiation of hippocampal NSCs, mimics and inhibitors were applied to transfected NSCs to alter expression. Real-time PCR was used to detect the overexpression and inhibition efficiency. miRNA corresponding to miR-3559-3P and miR-6324 mimics was highly expressed in NSCs (Fig. 5, *A*–*D*) (*p* < 0.001); miRNA corresponding to miR-3559-3P and miR-6324 inhibitors was inhibited in NSCs (Fig. 5, *A*–*D*) (*p* < 0.001). The expression level of miR-3559-3P and miR-6324 in NSCs transfected with 1 nM miRNA mimics was similar to the expression pattern of miR-3559-3P and miR-6324 in NSCs cocultured with transected exosomes, two to threefold to the normal group.

**Figure 6:**
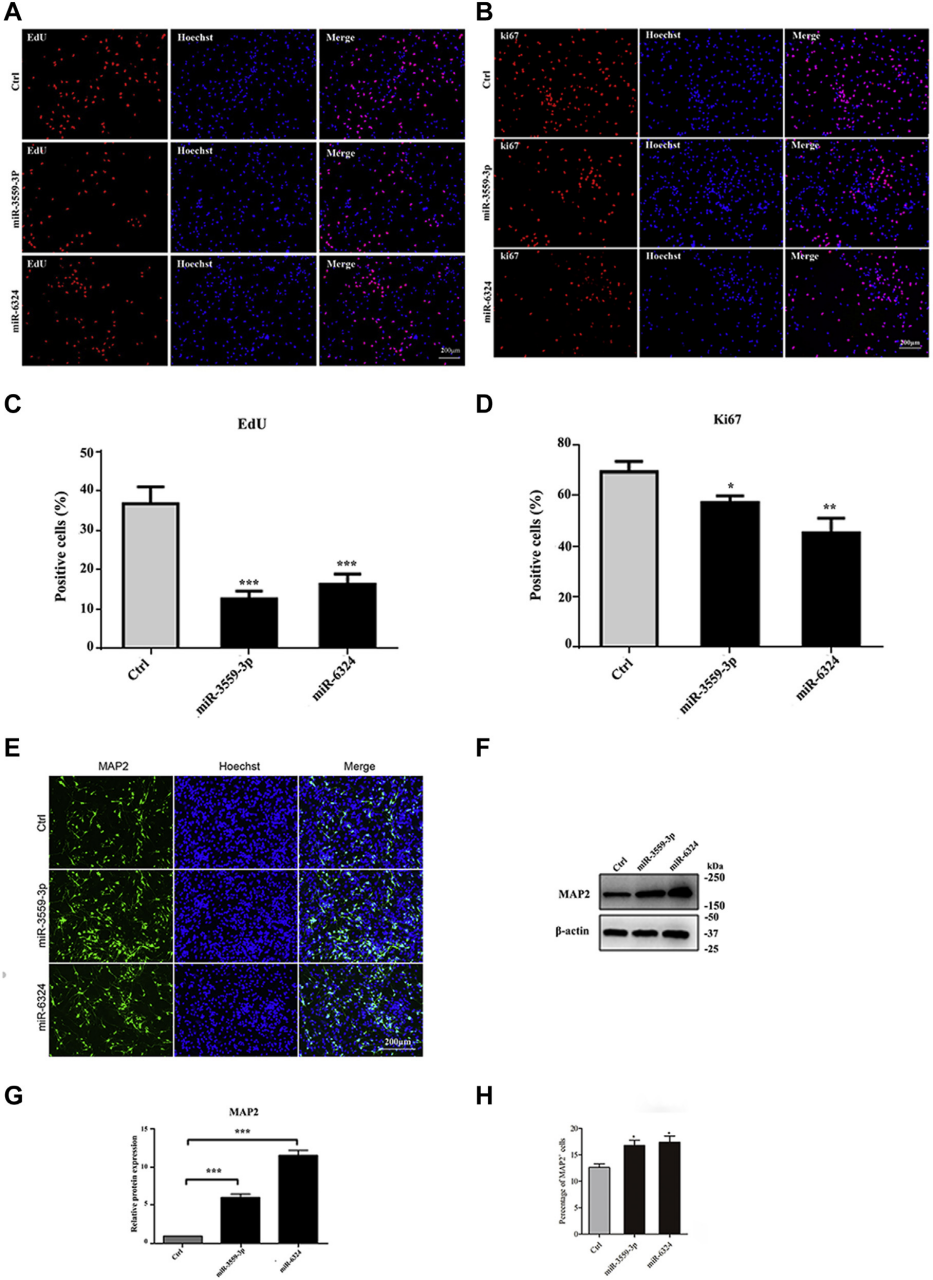
**Effect of miR-3559-3p and miR-6324 overexpression on proliferation and differentiation of NSCs.***A*, expression in EdU-positive cells. *B*, expression in Ki67-positive cells. *C*–*D*, statistical analysis showed that the expression in EdU- and Ki67-positive cells in the experimental group was significantly decreased compared with the control group (∗*p* < 0.05, ∗∗*p* < 0.01, ∗∗∗*p* < 0.001). *E*, MAP2-positive cell changes. *F*, MAP2 expression levels. *G*–*H*, statistical analysis showed that MAP2 expression levels and MAP2-positive cells were upregulated (∗*p* < 0.05, ∗∗*p* < 0.01, ∗∗∗*p* < 0.001).

Real-time PCR also showed that the expression level of the neuron-specific molecules MAP2, SYN1, NEUROD1, and STMN2 increased significantly when NSCs were transfected with 1 nM miRNA mimics for 24 h (Fig. 5, *E* and *G*); the expression of these four molecules decreased significantly when NSCs were transfected with 20 nM miRNA inhibitors for 24 h (Fig. 5, *F* and *H*). The EdU assay showed that the percentage of EdU-positive cells was lower in NSCs overexpressing miR-3559-3P group than the control group. The percentage of Ki67-positive cells was also lower in NSCs overexpressing miR-3559-3P group than the control group. In addition, the percentage of NSCs overexpressing miR-6324 was lower in the EdU-positive group than the control group; the percentage of NSCs overexpressing miR-6324 was also lower in the Ki67-positive group than the control group (Fig. 6, *A*–*D*). Immunofluorescence showed that the number of NSCs overexpressing miR-3559-3P was significantly higher in the MAP2-positive group than the control group. Western blots also showed that the expression of MAP2 was significantly upregulated in the group of NSCs overexpressing miR-3559-3P (Fig. 6, *E* and *G*) (*p* < 0.05 or 0.01). The percentage of NSCs overexpressing miR-6324 was lower in the EdU-positive group than the control group. The percentage of NSCs overexpressing miR-6324 was also lower in the Ki67-positive group than the control group. Immunofluorescence showed that the number of NSCs overexpressing miR-6324 was significantly higher in the MAP2-positive group than the control group. Western blots also showed that the expression of MAP2 was significantly upregulated in the group of NSCs overexpressing miR-6324 (Fig. 6, *F* and *H*) (*p* < 0.05 or 0.01).

### The effect of downregulation of miR-3559-3P and miR-6324 on proliferation and differentiation of hippocampal NSCs

**Figure 7:**
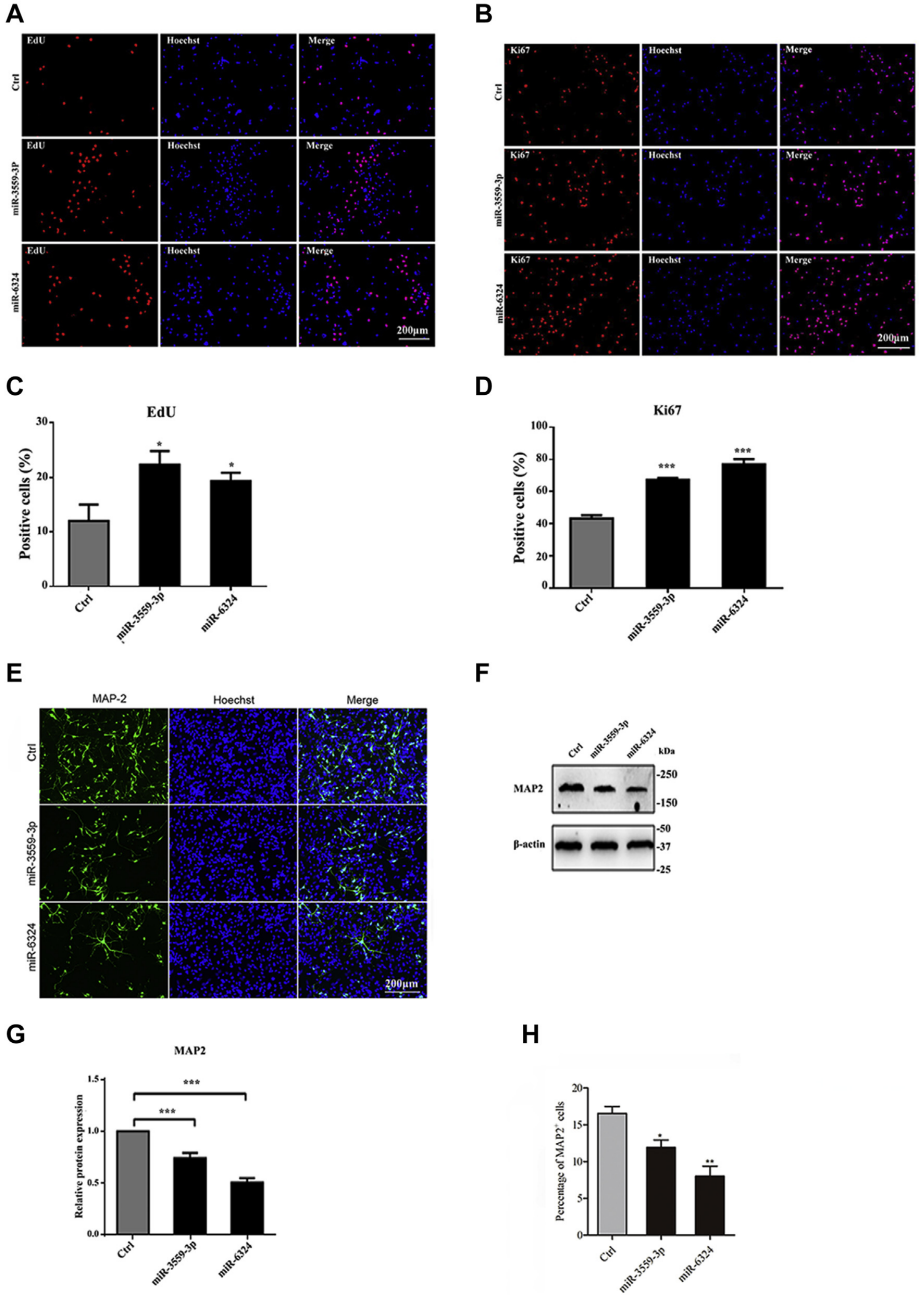
**Effect of miR-3559-3p and miR-6324 suppression on proliferation and differentiation of NSCs.***A*, expression in EdU-positive cells. *B*, expression in Ki67-positive cells. *C*–*D*, statistical analysis showed that the expression of EdU- and Ki67-positive cells in the experimental group was significantly increased compared with the control group (∗*p* < 0.05, ∗∗*p* < 0.01, ∗∗∗*p* < 0.001). *E*, MAP2-positive cell changes. Note: The control group in loss-function assay was the same as the control group in gain-function assay. *F*, MAP2 protein expression levels. *G*–*H*, statistical analysis showed that MAP2 expression levels and MAP2-positive cells were downregulated (∗*p* < 0.05, ∗∗*p* < 0.01, ∗∗∗*p* < 0.001.

The EdU assay showed that the percentage of NSCs with miR-3559-3P downregulated was higher in the EdU-positive group than the control group. The percentage of NSCs with miR-3559-3P downregulated was also higher in the Ki67-positive group than the control group. In addition, the percentage of NSCs with miR-6324 downregulated was higher in the EdU-positive group than the control group. The percentage of NSCs with miR-6324 downregulated was also higher in the Ki67-positive group than the control group (Fig. 7, *A*–*D*). Immunofluorescence showed that the number of cells with miR-3559-3P downregulated was significantly lower in the MAP2-positive group than the control group (Fig. 7, *E* and *H*). Western blots also showed that the expression of MAP2 protein was significantly downregulated in the group of NSCs with downregulated miR-3559-3P (Fig. 7, *F* and *G*) (*p* < 0.05 or 0.01). The percentage of NSCs with downregulated miR-6324 was higher in the EdU-positive group than the control group. The percentage of NSCs overexpressing miR-6324 was also lower in the Ki67-positive group than the control group. Immunofluorescence showed that the number of cells overexpressing miR-6324 was significantly higher in the MAP2-positive group than the control group. Western blots also showed that the expression of MAP2 protein was significantly upregulated in the group of NSCs with overexpressed miR-6324 (Fig. 7, *E*–*H*) (*p* < 0.05 or 0.01).

### The effect of the loss-of-function miRNAs in exosomes on proliferation and differentiation of cocultured NSCs

**Figure 8:**
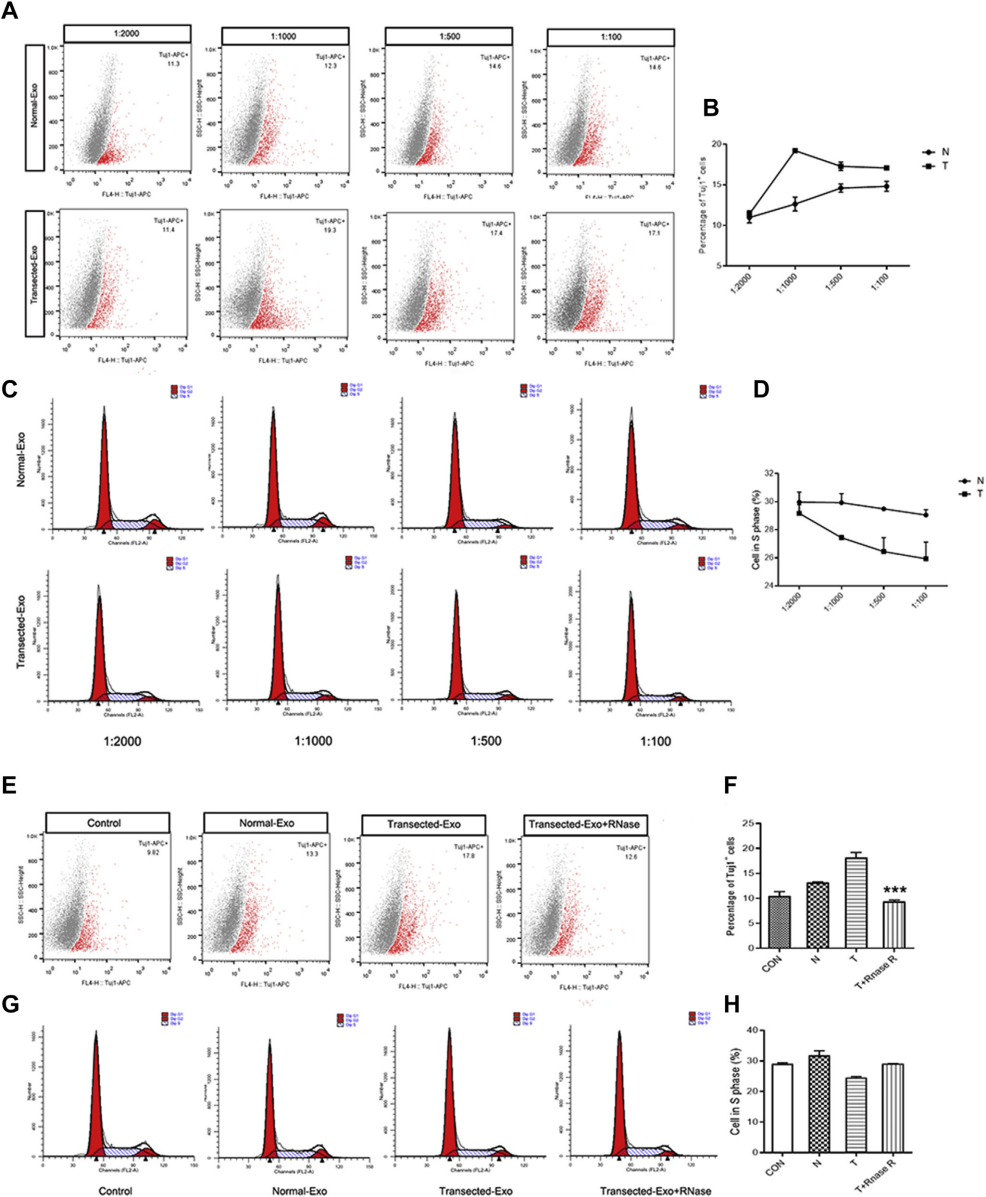
**The effect of the loss-of-function miRNAs in exosomes on proliferation and differentiation of cocultured NSCs.***A*, the percentage of Tuj1-positive cells in NSC differentiation at different ratio. *B*, statistical analysis showed that the percentage of Tuj1-positive cells in transected-Exol group at ratio of 1:1000 was significantly increased compared with the normal-Exol group. *C*, the percentage of cells in S phase in NSC proliferation at different ratio. *D*, statistical analysis of cells in S phase in each group for NSC proliferation. *E*–*F*, the FCM analysis showed that the percentage of Tuj1 positive cells in transected-Exo+RNase group was lower than that of transected-Exo group in differentiation assay (∗∗∗*p* < 0.001). *G*–*H*, the FCM analysis showed that the percentage of cells in S phase in each group in loss-of-function assay.

To verify the effect of miRNAs in exosomes on proliferation and differentiation of cocultured NSCs, the loss-of-function assays were performed. The percentage of Tuj1 positive cell in differentiation assay was the highest when NSCs were cocultured with transected exosomes at ratio of 1:1000 (Fig. 8, *A*–*B*), and the proliferation ability of cocultured NSCs decreased significantly at ratio of 1:1000 (Fig. 8, *C*–*D*). In loss-of-function assay, the miRNAs in exosomes were inactivated by the treatment of Triton-X100 and RNase. The results showed that the percentage of TUJ1 positive cells in transected-Exo+RNase group was lower than that of transected-Exo group in differentiation assay (Fig. 8, *E*–*F*). In proliferation assay, flow cytometry revealed that there was no significant difference of the percentage of S phase cells in each group (Fig. 8, *G*–*H*), implying that transected exosomes have no effect on the proliferation state of cocultured NSC after inactivation of miRNAs in exosomes.

## Discussion

Neural plasticity, the ability of the CNS to self-repair structure and function after damage strongly relies on neurogenesis (18, 19). The SGZ of the dentate gyrus in the hippocampus has the potential for neurogenesis throughout the entire adult period of life (20). Our previous study showed that the hippocampal niche dynamic changed after FFT. The exosome is a subtype of EV that possesses unique structural characteristics and biological functions. They can transfer mRNA, miRNA, lncRNA, circRNA, protein, and lipid between cells and are considered to be key messengers for short- and long-distance intracellular communication (12, 21). Considerable evidence supports their potential role in adult neurogenesis.

Neurons (22, 23) and glial cells (24, 25) can release exosomes, which can be collected from cerebrospinal fluid. These exosomes can transmit messages between homologous or heterologous cells. In this study, we obtained EVs from the rat hippocampus niche using the method of Vella *et al.* (26) Compared with the ultracentrifugation method, this method minimizes EV damage; however, the purity of the exosomes is slightly lower. Our results showed that the main peak particle size as measured by nanoparticle tracking analysis was slightly large (146.5 nm). The reason for this may be that the exosome diameter increased slightly after cryopreservation.

There are four possible mechanisms of cell communication involving exosomes: 1) a ligand on the exosome membrane directly binds to the corresponding receptor on the surface of the target cell membrane to activate intracellular signal transduction (juxtacrine mode); 2) an exosomal membrane protein is first cleaved by a protease in the extracellular space and a cleaved polypeptide fragment acts as a ligand that binds to a receptor on the surface of the target cell membrane to stimulate a signal pathway; 3) the exosome fuses with the target cell membrane to release its molecules into the cell in a nonselective manner; 4) the exosome may play a role in the formation of target cell multivesicular bodies by endocytosis (27, 28, 29, 30). In our study, a large number of CM-Dil labeled exosomes appeared in the cytoplasm of NSCs, suggesting that endocytosis may be the mechanism of entry into NSCs.

Two exosomal miRNAs closely related to neurodevelopment, miR-3559-3P and miR-6324, were selected by RNA sequencing based on previous functional studies. Overexpression of miR-3559-3P and miR-6324 in hippocampal NSCs promoted their differentiation into neurons while inhibiting their proliferation ability; in contrast, inhibition of miR-3559-3P and miR-6324 reduced differentiation into neurons. This result is consistent with previous studies that found the hippocampus niche is favorable for neurogenesis after denervation.

Adult neurogenesis is a multistep process comprising activation of quiescent aNSCs, their differentiation into committed progenitor cells, neuronal survival, migration, and functional integration of newborn neurons. miRNAs are an integral part of the gene regulatory networks driving these changes. Pons-Espinal *et al.* (31) focused on a pool of 11 miRNAs (including miR-124 and miR-134) that was found to be highly upregulated during early neuronal differentiation and asked whether these miRNAs could rescue the bias toward astrocytic differentiation and the impaired neuronal differentiation of Dicer cKO aNSCs. However, some of the miRNA functions might be context-dependent. For instance, miR-9 was found to maintain quiescence of aNSCs in the zebrafish brain (32), while in mouse aNSCs it promotes neuronal differentiation of aNSCs (33, 34). These divergent observations might be due to the different experimental model systems employed but may also reflect two different modes of action of miR-9, *i.e.*, cytoplasmic miR-9 promotes neuronal differentiation *via* canonical targeting of Tlx and Foxo1 (33, 34), while nuclear miR-9 maintains aNSC quiescence (35). Another example of a miRNA eliciting context-dependent effects is miR-137, which was found to promote differentiation of embryonic NSCs (35) but inhibits differentiation of adult NSCs (36). miR-3559-3P and miR-3552 from adult neurogenic niche might also act in a context-dependent manner. Thus, elucidating the differences in miRNA-based regulation of murine *versus* human adult neurogenesis might eventually enable the promotion of adult neurogenesis in humans and their exploitation for regenerative purposes.

There are still several issues to be further explored. We should clarify the mRNAs involved in regulation of NSC proliferation and differentiation that are targeted by miR-3559-3P and miR-6324. In addition, the relevant signaling pathway still needs to be elucidated, as well as the effect of these two miRNAs on NSC differentiation into neurons of specific phenotypes (such as cholinergic neurons). Clarifying these issues will provide clues and new ideas for uncovering the NSC differentiation mechanism and applying exosomes in the treatment of neurodegenerative diseases.

## Experimental procedures

### Animals

In total, 25 adult Sprague-Dawley rats (12 male and 13 female) and 8 E17 Sprague-Dawley rats were used in this study. All animals were provided by the Experimental Animal Centre of Nantong University, China. Experimental procedures were conducted according to the Jiangsu Institutes of Health Guide for the Care and Use of Laboratory Animals. All efforts were made to minimize the number and suffering of animals used in the study. The rats were housed and caged at room temperature (23 °C ± 2 deg. C) in 12 h light/12 h dark cycle conditions in a facility where food and water were available. The rats were anesthetized with chloral hydrate (2 ml/kg) for FFT, which was performed with a wire knife at the CA1 layer of the dorsal hippocampus at the following coordinates based on the bregma: anteroposterior, 1.4 mm; lateral, 1 to 4 mm; depth, 5.6 mm. There was no restriction on the gender of the animals.

### Extraction of exosomes from the hippocampal niche after FFT

The rat hippocampal tissue (denervated and untreated) was quickly harvested on ice. It was then digested with 0.125% trypsin at 37 °C for 30 min and the serum without exosomes was added to stop digestion. The Total Exosome Isolation Kit (from plasma) (Invitrogen, USA) was added to the supernatant after 4 °C, 3000*g*, 30 min centrifugation for an overnight stand. Then, after 4 °C, 10,000*g*, 1 h centrifugation, the precipitation was resuspended with 50 μl DPBS, and exosomes were harvested for the following experiments.

### Western blot

Lysates of hippocampi and cells from each group were separated on 10% SDS-polyacrylamide gel and transferred to a nitrocellulose membrane for identification of exosome markers (CD9, CD63, and Alix) and neuron-specific molecules (MAP2 and Ki67). The membrane was blocked in TBST containing 5% nonfat milk for 1 h and incubated with rabbit anti-CD9 (1:800 Abcam), rabbit anti-CD63 (1:800 Abcam), mouse anti-CD63 (1:1000 Abcam), mouse anti-MAP2 (1:800 Abcam), rabbit anti-Ki67(1:200 Abcam), and mouse anti-β-actin (1:10,000 Sigma) antibodies in TBST/5% BSA overnight. After washing, the membrane was incubated for 2 h with goat peroxidase-conjugated IgG diluted 1:5000 in TBST/5% BSA. Immunoreactive bands were visualized with the ChemiDocTM Touch Imaging System (Bio-Rad).

### TEM assay

A 10 μl suspension of exosomes was diluted into a suitable concentration with DPBS. A drop of exosome suspension was placed on a copper net and allowed to stand for 5 min. Then, a drop of 1% dye solution of phosphotungstic acid was added. The copper net was dried under an infrared baking lamp for 10 min prior to sample examination with a transmission electron microscope.

### RNA sequencing

The exosomes from hippocampal niche were collected, pooled together, and lysed in Trizol reagent, and then total RNA was extracted. After evaluation of the RNA quality, the qualifified samples were submitted to a Sangon (China, Shanghai) for high-throughput sequencing.Samples with a high RNA integrity number (>8) were used for library construction. One hundred nanograms of total RNA was used for each sequencing library. All libraries were then sequenced using the Illumina HiSeq 2000 Sequencer. Two replicates of samples were sequenced. The differentially expressed genes were selected by the following criteria: fold change no less than 2, and FDR value less than 0.01.

### Bioinformatics analysis

Pathway significant enrichment analysis was based on the KEGG Pathway Public database and found that the significant enrichment pathway among the differential expressed genes applied the hypergeometric test. With the pathway analysis results, we can identify the main biological process and signal transduction pathways that the differential expressed genes involved.

### Cell transfection

The transfection of NSCs was carried out using Lipofectamine 3000 (Invitrogen). The miRNA mimics and inhibitors and negative control for transfection were synthesized by RiboBio (China, Guangzhou). The NSCs were transfected with a 1 nM miRNA mimics using Lipofectamine 3000 following the manufacturer’s instructions to simulate the expression pattern of miRNAs in NSCs after intaking the exosomes. The NSCs were transfected with a 20 nM miRNA inhibitors using Lipofectamine 3000 following the manufacturer’s instructions. After transfection for 48 h, the expression levels of the selected lncRNAs were measured by qPCR. After 72 h, the cells were collected for cell proliferation assays and differentiation assay.

### Real-time PCR

Three rats in each group were used for real-time PCR for each experiment. Total extracted RNA was quantified and quality checked using the Nanodrop 2000 spectrophotometer (Thermo Scientific, USA). Transcripts were reverse transcribed using the RevertAid First Strand cDNA Synthesis Kit (Thermo Scientific, USA) at 65 °C for 5 min, 42 °C for 60 min, and 72 °C for 5 min. The sequence of primers used for PCR gene amplification was as follows: Stmn2 sense 5′-CACTTGGAGACTGTGAGCTGGTT-3′ and antisense 5′-ATTGCCCTATGGGAATGAAA-3′; Neurod1 sense 5′-CAGGGTTATGAGATCGTCACTATTC-3′ and antisense 5′-CCTTCTTGTCTGCCTCGTGTTCC-3′; Map2 sense 5′-CTTGATTCTATTGCCCTTGGGTTTA-3′ and antisense 5′-CATCCATCGTTCCGCTAGTGTTG-3′. Primers of miRNAs were designed and synthesized by RiboBio (Guangdong, China). The intellectual property rights of the primer sequence belong to Ribo biology, which were asked to be classified. Quantitative real-time PCR was conducted using SYBR Green Master Mix (Roche, Germany). PCR reactions were performed at 95 °C for 40 s, 59 °C for 40 s, and repeated for 40 cycles. GAPDH and U6 were used as endogenous controls. Fold changes were calculated using the relative quantification 2^−ΔΔCt^ method. All experiments were performed in triplicate.

### Coculture assay

The harvested exosomes were labeled with celltracker CM-Dil following the manufacturer’s instruction (YEASEN, China). Then, the labeled exosomes were cocultured with 5 × 10^4^ NSCs for 48 h. Positive NSCs were detected by the live cell station.

### Cell proliferation assay

NSC proliferation was measured using the EdU (5-Ethynyl-2’-deoxyurdine) assay. Harvested NSCs were incubated in a 1.5 ml centrifuge tube for 2 h at 37 °C and mixed with 4% formaldehyde for 30 min at room temperature. After washing twice with 1 ml of PBS, NSCs were stained with Apollo 567 solution for 30 min at room temperature and Hoechst 33342 solution for 30 min and visualized using a fluorescent microscope (Olympus). The EdU incorporation rate was expressed as the ratio of EdU-positive cells (red cells) to total Hoechst 33342-positive cells (blue cells).

### FCM assay

Cell suspension with PBS was subjected to flow cytometry. Cells in a culture dish were trypsinized and collected, washed twice in PBS, and fixed in 1× Fix/Perm Buffer working solution at 4 °C for 40 min. After washing with 1× Perm/Wash Buffer, cell samples were mixed with 100 μl of 1× Perm/Wash Buffer and incubated with APC-conjugated anti-Tuj1 antibody (BD Biosciences) at 4 °C for 2 h. Cell suspension was subjected to flow cytometry. In proliferation assay, cells were fixed overnight at 1 × 10^6^ cells/ml in precooled 75% alcohol after dispersion with trypsin (Sigma) and filtration through a 40-μm cell strainer. Cells were then harvested and stained with Annexin V-fuorescein isothiocyanate and propidium iodide (PI) (BD Biosciences, USA) for 30 min following manufacturer’s instructions. Cells were analyzed using flow cytometry (BD Biosciences, USA). Data were analyzed using the CELL Quest 3.0 software. All experiments were performed in triplicate.

### Statistical analysis

Each experiment was confirmed using three independent biological replicates, unless otherwise specified. All analyzes were conducted using SPSS 19.0 statistical software (SPSS, IL, USA). Data are expressed as means ± standard deviation. Statistical significance was determined using the Student’s t test. *p* < 0.05 was considered significant.

## Data availability

All are included in this article.

## Conflict of interest

The authors declare that they have no conflicts of interest with the contents of this article.
